# The diversity of providers’ and consumers’ views of virtual versus inpatient care provision: a qualitative study

**DOI:** 10.1186/s12913-023-09715-x

**Published:** 2023-07-04

**Authors:** Robyn Clay-Williams, Peter Hibbert, Ann Carrigan, Natalie Roberts, Elizabeth Austin, Diana Fajardo Pulido, Isabelle Meulenbroeks, Hoa Mi Nguyen, Mitchell Sarkies, Sarah Hatem, Katherine Maka, Graeme Loy, Jeffrey Braithwaite

**Affiliations:** 1 Australian Institute of Health Innovation, Centre for Healthcare Resilience and Implementation Science, Macquarie University, Sydney, NSW Australia; 2 IIMPACT in Health, Allied Health and Human Performance, University of South Australia, Adelaide, SA Australia; 3 Centre for Elite Performance, Macquarie University, Expertise & Training, Sydney, NSW Australia; 4 Western Sydney Local Health District, Sydney, NSW Australia

**Keywords:** Virtual care, Consumers’ views of care, Providers’ views of care, Qualitative research, Hospitals, Innovation in care models

## Abstract

**Background:**

A broad-based international shift to virtual care models over recent years has accelerated following COVID-19. Although there are increasing numbers of studies and reviews, less is known about clinicians’ and consumers’ perspectives concerning virtual modes in contrast to inpatient modes of delivery.

**Methods:**

We conducted a mixed-methods study in late 2021 examining consumers’ and providers’ expectations of and perspectives on virtual care in the context of a new facility planned for the north-western suburbs of Sydney, Australia. Data were collected via a series of workshops, and a demographic survey. Recorded qualitative text data were analysed thematically, and surveys were analysed using SPSS v22.

**Results:**

Across 12 workshops, 33 consumers and 49 providers from varied backgrounds, ethnicities, language groups, age ranges and professions participated. Four advantages, strengths or benefits of virtual care reported were: *patient factors and wellbeing, accessibility, better care and health outcomes,* and *additional health system benefits,* while four disadvantages, weaknesses or risks of virtual care were: *patient factors and wellbeing, accessibility, resources and infrastructure*, and *quality and safety of care*.

**Conclusions:**

Virtual care was widely supported but the model is not suitable for all patients. Health and digital literacy and appropriate patient selection were key success criteria, as was patient choice. Key concerns included technology failures or limitations and that virtual models may be no more efficient than inpatient care models. Considering consumer and provider views and expectations prior to introducing virtual models of care may facilitate greater acceptance and uptake.

**Supplementary Information:**

The online version contains supplementary material available at 10.1186/s12913-023-09715-x.

## Background

Virtual care is broadly defined as services delivered remotely from patients [1]. The virtual care model of healthcare delivery typically takes one or more of four forms: patient care and consultation delivered through telephone or video communication; remote monitoring of patients’ condition or symptoms; transmission of health related information such as electrocardiograms (ECG/EKG) over telephone or internet; and provision of specialist advice over telephone or internet to clinicians working remotely, in rural or regional locations [2]. Virtual care interventions, such as telehealth or remote monitoring, have been implemented in multiple settings across chronic and acute conditions, including: heart failure, chronic obstructive pulmonary disease (COPD), asthma, chronic kidney disease (CKD), fractures, myocardial infarction, and postnatal depression [3]. Virtual care has been shown to reduce costs [4], increase consumer-managed care and self-monitoring outcomes (including diet, inhaler and medication adherence) [5–10], and improve patient knowledge [7, 11–13] and satisfaction [11, 14–16]. For some conditions, delivery of care via virtual modes has also been shown to reduce hospital readmissions [17, 18] and patient mortality [19–21], and improve clinical indicators [12, 15, 19, 22] and healthcare related quality of life [23–26].

Uptake of virtual care has increased over the last decade alongside improved capability of technology, such as availability of, and access to, high-speed broadband internet. Delivery of healthcare through virtual modes expanded rapidly with the advent of the COVID-19 pandemic, in response to demand, to reduce pressure on hospitals, and to mitigate risks of virus transmission [27]. In a recent umbrella review on seven innovative models of healthcare, for example, 43 out of 61 reviews reported on the virtual care model [3]. Thirty-five of these reviews compared virtual modes to usual care, while eight reviews compared virtual care with other models such as ambulatory care, digital hospital, hospital in the home, integrated care, and specialist hospitals. While this may suggest that virtual care interventions have a strong evidence base in the literature, it may be partially attributable to the volume of published studies on virtual care associated with the pandemic. It is likely that some innovations from the pandemic, such as some uses of telehealth and virtual care, did not uniformly deliver better-value care to all patients [28].

As the demands of COVID-19 subside, it is timely to examine whether virtual care should continue to hold a prominent position in a hospital’s strategy for care delivery; in particular, whether it is perceived by clinicians, and the patients they treat, to provide what is needed for their healthcare. Recent research highlighted the importance of moving away from an emphasis on technology toward a consumer focus model that includes engaging with patients to design virtual care to better meet their needs [29]. Our study therefore sought to elicit consumers’ and providers’ views and preferences about provision of care via virtual modes. We built the study around the design of a proposed new metropolitan hospital in a large diverse catchment in New South Wales (NSW), Australia, but with generalised application of the findings to similar health systems internationally. It was part of a larger study to examine strategies for implementing innovative models of care in new hospitals [30].

## Methods

We designed and executed a mixed-methods study of consumers’ and providers’ needs and expectations in relation to innovative models of care delivery for a new health facility. Study methods are described in detail elsewhere [30]. In this paper we report the methods and results specific to the virtual model of care.

### Study design

The study design was underpinned by an academic literature review of the international evidence supporting the efficacy of virtual care. Consumer and provider demographic information, including digital literacy levels, were collected during the recruitment process via a short expression of interest (EOI) questionnaire and the Participant Information and Consent Form (PICF). Consumer and provider perceptions on strengths and benefits, barriers, enablers, and safety and risks associated with provision of care through virtual modes were collected in facilitator-coordinated workshops.

### Study setting

Workshops were conducted online, via the Zoom platform. Participants were provided with options to attend 2-h workshops during, or outside of, working hours.

### Participants

Consumers included residents and patient representatives within the new health facility catchment, comprising 49 suburbs in Sydney, New South Wales, Australia. The facility catchment area was defined by the Local Health District’s (LHD) planning team on 16^th^ July 2021. Participants were recruited through the LHD’s consumer and provider networks via email, postings in local newspapers and through advertisements on the LHD’s Facebook page. As 37% of the consumers in the catchment area are from culturally and linguistically diverse (CALD) backgrounds [31], non-English speaking consumers were invited to participate in the study through advertisements translated into the four most common languages spoken in the area (Punjabi, Hindi, Mandarin and Korean). Their participation was aided by bi-lingual interpreters from the LHD.

The study invitation included a link to an online EOI questionnaire using REDCap electronic data capture tools [32]. Demographic data collected in the questionnaire included age, gender, location, ethnicity and contact information. Providers were asked to indicate their role and specialty, and consumers were asked for pertinent health information such as whether they are experiencing a chronic health condition. Response to the questionnaire was taken as implied consent for collection of the demographic information. Interested consumers and providers were invited to participate in one of a series of workshops, run over a six-week period. Participants were consented via a separate PICF prior to each workshop; digital literacy questions in the PICF included eliciting participant familiarity and confidence with using smart phones, smart watches, and computers.

### Workshops

Virtual care was presented and discussed in 12 workshops; six for consumers and six for providers. One of the consumer workshops was designated for CALD participants, and conducted with the assistance of Mandarin speaking bi-lingual interpreters; one of the provider workshops was specifically conducted for primary care physicians (General Practitioners; GP) to leverage the considerable experience accumulated in telehealth delivery by this group over the preceding two years. The workshops commenced with a short explanation by a research team lead about the purpose of the workshop. Researchers (one scribe and one facilitator), and participants were then allocated to smaller online focus groups of up to five people. Within each group, the researchers made notes, facilitated discussion, and asked probing questions (facilitator guide available on request). Audio-recording devices, and researcher notes were used to capture the content of discussions.

**Fig. 1:**
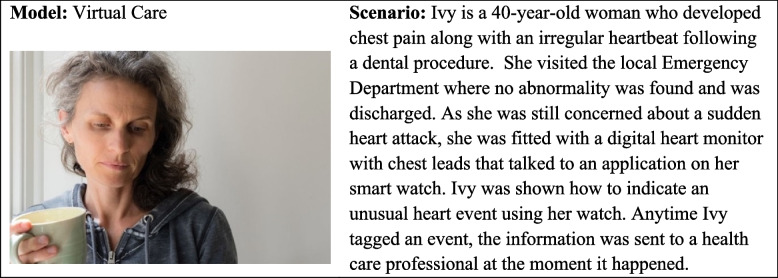
Scenario for virtual care

Workshop scenarios and questions were designed around the model of care to provide an example. Chest pain was the condition used as heart disease is one of the most common reasons for hospitalisation identified in the health facility catchment (see Fig. 1). As participants were likely to be familiar with care delivered over the telephone or via video, due to the high prevalence of this care delivery mode during COVID-19, the scenario illustrated virtual care in the form of remote monitoring. We asked participants general questions about the model’s strengths and weaknesses, usability and safety for themselves and people in their care. For providers, we also asked about barriers and enablers that might be encountered if introducing the model, from their own and their patients’ perspectives. Scripts for the workshops are provided as Supplemental files #1 (consumer) and #2 (provider).

### Data analysis

Data from the demographic questionnaire were descriptively analysed using SPSS V.22.0. Consumer and provider workshop data were de-identified and merged into aggregated narrative summaries, one for consumers and one for providers, and analysed separately. Two researchers (AC, NR) independently conducted a thematic analysis of the data using an open coding process then, through discussion, merged codes to derive sub-themes, which were then grouped into key themes that represented consumer and provider views and preferences about provision of care via virtual modes.

## Results

### Participants

A total of 33 consumers and 49 providers participated in the 12 workshops where virtual care was considered. Their age and gender distributions are summarised in Table 1. Focus groups were conducted for all consumers and providers who expressed an interest in participating in the study.

**Table 1:** Consumer and provider participant demographics

	**Consumers (*n*)**	**Providers (*n*)**
Gender		
Male	13	15
Female	20	34
Age		
Under 30	3	10
31 to 45	5	19
46 to 60	14	15
Over 61	10	5
Prefer not to say	1	0

Consumers reported experiencing health conditions that were spread across the major physiology systems, with acute or chronic cardiac, renal or bone related conditions most common. This was representative of the catchment where chest pain, heart failure and acute myocardial infarction are listed among the most common causes of hospitalisation [33].

Most of the consumers rated their proficiency in English as excellent or good, (73%) although around half of the participants (48%) reported speaking another language at home. Almost half of the consumers identified as Australian (49%) and there was evidence of ethnic diversity (see Table 2).

**Table 2:** Participant ethnicity and other languages spoken at home

Ethnicity^c^ (%)		Other language spoken at home^d^ (%)	
**Consumers**	**Providers**	**Consumers**	**Providers**
Australian (49)	Australian (62)	Mandarin (65)	Mandarin (17)
Chinese (30)	Asian (14)	Hindi (6)	Hindi (11)
Indian (8)	Indian (5)	Tamil (6)	Italian (11)
European (4)	Middle Eastern (3)	Croatian (6)	Sinhalese (11)
Middle Eastern (3)	European (3)	Serbian (6)	Afrikaans (6)
Fijian Indian (3)	South American (3)	Tagalog (6)	Maltese (6)
Asian (3)	Aboriginal and Torres Strait Islander (3)	Punjabi (6)	Gujarati (6)
	Other^a^ (8)		Other^b^

^a^ Includes North American (2), New Zealander (2), Filipino (2), and Sri Lankan (2)

^b^ Includes Japanese (6), Polish (6), Slovenian (6), Spanish (6), Arabic (6), Cantonese (6)

^c^ Participants were able to select more than one option

^d^ Columns do not add up to 100 due to rounding

**Fig. 2:**
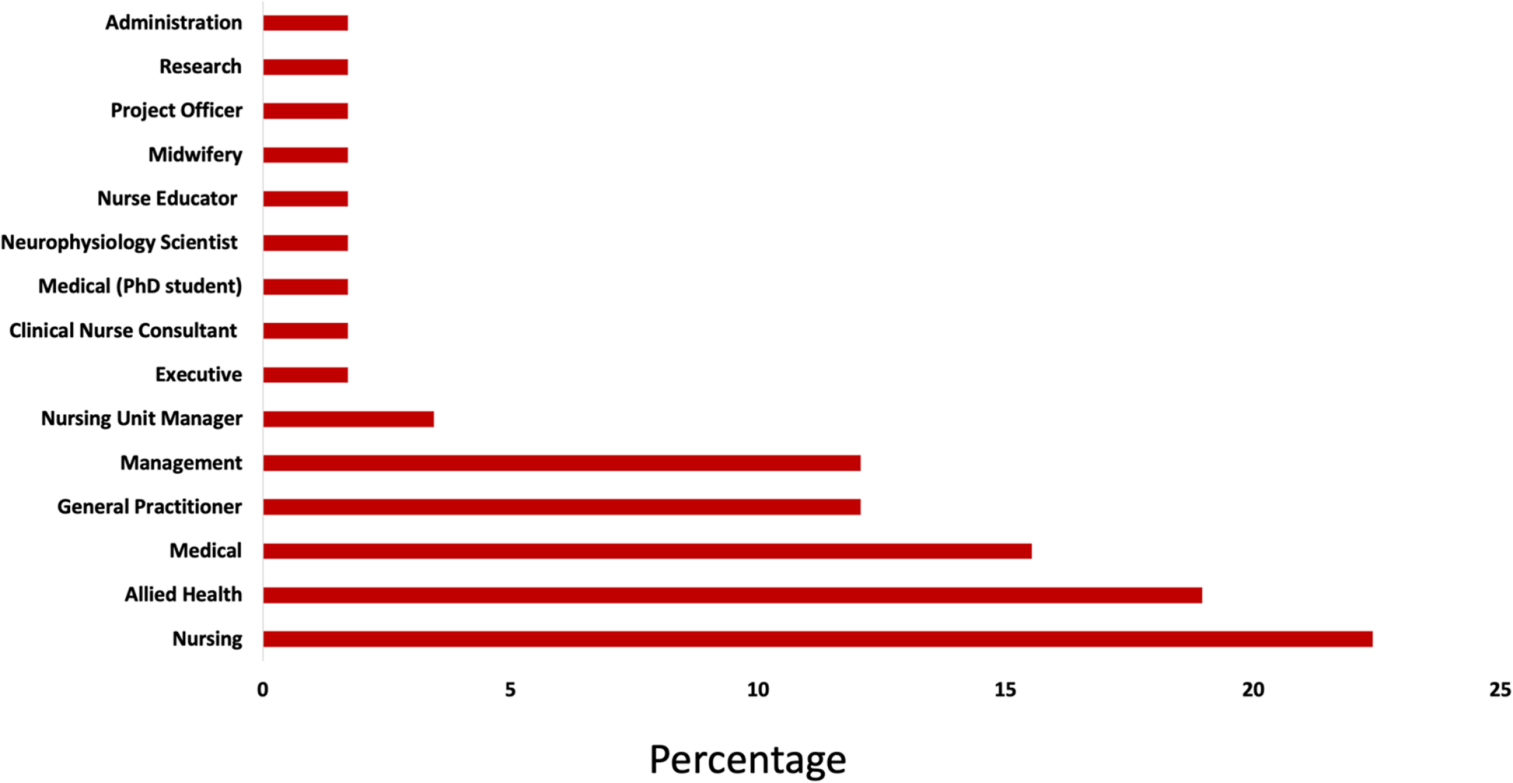
Distribution of workplace roles of provider participants

For providers, 47% worked in the LHD while the remaining 53% worked outside the LHD but resided in the new hospital catchment area. The providers worked in a variety of professional roles including nursing, allied health (e.g., physiotherapy, speech pathology), medical, general practice and administration (see Fig. 2). The providers reported having a diverse range of medical specialist qualifications with most practicing in a speciality such as psychiatry (25%), bone (16%), lung (15%), abdominal (13%), heart (12%), postnatal depression (10%), or renal dialysis (9%).

All providers self-rated their English proficiency as excellent or good, with 37% speaking another language at home. Although most of the providers identified as Australian (62%), there was evidence of ethnic diversity (see Table 2).

### Workshops

The two main forms of the virtual care model that were discussed within workshops were remote monitoring, and telehealth or videoconferencing. As expected, most consumers and healthcare providers were familiar with a consultation version of the virtual care model due to their experience with COVID-19, where it was heavily utilised in primary care in NSW. Consumers and providers appeared to have a common understanding of the broad concepts associated with different forms of virtual care but, while consumers tended to reflect on the model from a standpoint of patient needs, providers were able to consider both provider and patient views. CALD participants raised similar issues to other consumers but emphasised the need for communication between provider and consumer in a common language: *“From working as a language translator, language is an issue, can be a severe issue – how to convert the language*” (CALD Consumer 1, workshop 10).

#### Advantages of virtual care

The four themes that emerged from both consumer and provider data on the various strengths and benefits of hospital virtual care were *patient factors and wellbeing, accessibility, better care and health outcomes,* and *additional health system benefits* (see Table 3). Across the workshops, consumers and providers described virtual care as an accessible, patient-centred model that could provide agency and reassurance for patients in the community. It was perceived by consumers as a convenient way to receive care, as it avoided disruption and travel (particularly for those in more rural areas). The model was seen as a good alternative to inpatient care, particularly for patients with stable, low acuity conditions that required ongoing medical monitoring. GPs felt that ongoing monitoring could provide them with additional insights into their patient’s condition, contributing to bridging the gap between primary and tertiary care. Providers indicated a broad scope for the applicability of virtual care, particularly for patients who do not have complex needs, and felt that with appropriate triage processes, staff could treat most patients remotely. Virtual care was also seen as a model that could reduce pressure on hospitals by freeing up resources, such as beds, and ameliorate the risk of infection associated with hospitalisation.

**Table 3:** Strengths and benefits of virtual care

Strengths and benefits	Illustrative quote(s)
**Patient factors and wellbeing**	
Consumer perceptions: • Patient can be monitored at home, and avoid hospital with its inherent risks • Control and assurance, with instant feedback on own condition • Feelings of security and confidence in being monitored and having ongoing support	*“Peace of mind – someone sees straight away and can call for help” Consumer 1, workshop 6*
Provider perceptions: • Tailored to patient, with increased time under care, but decreased time in hospital • More comfortable and faster for patient, only need to visit hospital when needed • Easy to track patient appointments and movements, no need for patient to remember details if events auto-monitored • Decrease in anxiety, as patient is reassured and empowered	*“More comfortable for the patient to be at home as there is no hospital noise” Provider 2, workshop 2* *“Not worrying about having a heart attack and no one being aware of it” Provider 1, workshop 6*
**Accessibility**	
Consumer perceptions: • Easy to use, even for those with low digital literacy • Allows access to care while at home, including 24-h monitoring • Convenient, timely, less disruptive to daily routines • Allows follow-up care to be fast-tracked	*“Time saver for the patient – she doesn’t have to be in ED, she is at the comfort of her own home” Consumer 6, workshop 8* *“It will save time to communicate with specialists and have appointments with the doctor” Consumer 2, workshop 8*
Provider perceptions: • Easy, convenient, efficient and cheaper for consumers and providers • Better access, from home, to reliable and streamlined communication • Allows consumer to be regularly seen by specialist • Increases reach of care e.g., to rural and remote communities	*“Have capacity to provide care to those who are not close. It increases the reach of care” Provider 2, workshop 8* *“Improved convenience for both patients and providers. Can have an appointment, or through an online “virtual clinic” waiting room” Provider 6, workshop 6*
**Better care and health outcomes**	
Consumer perceptions: • Continuous, safer care at home, minimising potential for cross-infection • Patient will be able to escalate urgent care and communicate with healthcare staff • Improved insights with data capture at hospital and 24-h monitoring • Access to post-hospital care	*“It is better than the current system where you have to wait three months to be seen by the doctor with results” Consumer 1, workshop 8* *“Good to include this in the hospital because it will help to find out if the patient is severe or mild and then decide if they need to go to the hospital. Then when they are stable enough to go home, they can free up the beds for other patients” Consumer 1 workshop 2*
Provider perceptions: • Reliable and well-performing technology • Senior staff member reviews data • GP access to information will strengthen clinical decision making • Can monitor for extended period and capture real world data during activities, including logging events that are not captured during ED visit • A time-sensitive model • Reduces infection risk, as patient spends less time in hospital	*“From an ED point of view, it could be useful. It is hard to recreate SVT and other episodes – could log the number of times the SVT occurs” Provider 3, workshop 6*
**Health system benefits**	
Consumer perceptions: • Shortens the time spent as an inpatient • Reduce hospital pressure, by freeing up beds • Streamline ED triage process for low acuity patients, with less patients in waiting room	*“It would be a very good way to help the patient, as well as helping the hospital – lighten the burden of many patients by having patient at home” Consumer 7, workshop 2*
Provider perceptions: • Relieves bed pressure • Keeps people out of hospitals, and cuts numbers in clinics • Time efficient • Data can be readily available to teams	*“This care has been delivered for many years, it’s good for the patient in that they don’t have to always go to clinic, as long as there is a plan outlined for them like red flags and all that… from a cardiology specialist perspective they are getting care and have real time monitoring and if there is an abnormality they have options – win–win situation” Provider 1, workshop 2*

#### Disadvantages of virtual care

The four themes that emerged from both consumer and provider data on the barriers, difficulties and risks associated with virtual care were: *patient factors and wellbeing, accessibility, resources and infrastructure*, and *quality and safety of care* (see Table 4). Consumers and providers felt that patient wellbeing might suffer if patient characteristics, such as health and digital literacy or self-efficacy, and severity of the illness, were not adequately considered when allocating a patient to this mode of care. They also emphasised the importance of patient choice when utilising virtual care, and the potential for low quality care (e.g., lack of communication, inaccurate or invalid monitoring, and overreliance on technology), if the provider depends solely or mainly on technology over face-to-face care. Consumers raised concerns about the accessibility of the virtual care mode, particularly for patients with low socioeconomic backgrounds, poor health and digital literacy or no access to technology or equipment. Providers raised similar accessibility concerns to these but were also cognisant of potential barriers arising from costs of providing around-the-clock (24/7) care and provision of language translators. Resources and infrastructure concerns of both consumers and providers revolved around staffing, and availability and reliability of technology including WiFi and monitoring devices. Safety issues, such as data safety and privacy, the risk of hacking, and the need to develop escalation procedures for emergency care were raised by both groups. As a result, virtual care was often perceived as a complementary model or delivery mode to support other models of care, rather than as a standalone model.

**Table 4:** Barriers, challenges and risks of virtual care

Barriers, challenges, and risks	Example quote(s)
**Patient factors and wellbeing**	
Consumer perceptions: • Choice is important • May not be suitable for severe conditions e.g., heart condition vs. sleep monitoring • Timeliness of urgent care, and what to do in an emergency • Anxiety, if patient uncomfortable with self-monitoring, unfamiliar with clinicians, a mental health patient, living alone, or feeling like they should be in hospital • Fallibility of human follow-up • Lack of education about model, or information about condition and care plan • Inconvenience of carrying heavy and bulky monitoring devices • Model relies on patient disclosure • Impersonal care, concern that the assessment and diagnosis is not thorough enough, or patients feeling like they are being sidelined	*“If I was very sick, I wouldn’t want to use this model” Consumer 2 workshop 2* *“I feel like this model is better for people with less severe conditions or diseases. For people with more severe situations or diseases, you might miss the best timing to get treated” CALD Consumer 2, workshop 10* *“Relies on patient disclosure and ability to pick up problems with their health. It will be difficult for some patients, or some patients won’t be truthful” Consumer 2 workshop 8*
Provider perceptions: • Limits communication and face-to-face contact • Patient irresponsibility or lack of compliance e.g. patient forgets to use the device or does not follow up on care advice, or patients may try to manipulate data for nefarious purposes such as drug seeking • Characteristics of patients e.g. poor sensory ability, living alone with no carer, or anxious due to self-monitoring • May feel less supported, or lack trust in the system and privacy protections	*“Technology gives a false sense of security” Provider 1, workshop 2* *“There are some challenges with different cultures who would prefer face to face” Provider 2, workshop 2*
**Accessibility**	
Consumer perceptions: • Feeling disadvantaged if you do not use the model • Subject to availability of doctors and waiting times • Access to equipment e.g. technology may be expensive for health systems and consumers, leading to inequity of access • Education and various competencies needed e.g. time/education to learn how to use the device, English competency, digital literacy, health literacy of the patient or family • Not flexible for all patients	*“I would worry about older family members using this model unless they had support (carer or other person)” Consumer 1, workshop 2* *“It will be difficult for CALD patient learn how to use the device and interact with the technology” CALD Consumer 1, workshop 10*
Provider perceptions: • Cost to patient and health system, including maintenance of the model • Accessibility in a timely manner • May not be suitable for paediatric or geriatric patients, or those with low health or digital literacy • Cultural barriers, language and translation, and other considerations for CALD patients • Health inequity if patient cannot access internet and technology	*“Interpreters prefer video conferencing. You don’t have the visual information to allow for interpreting over the phone” Provider 10, workshop 6* *“Patients that are technologically disadvantaged are too scared and don’t understand how things work, rely on teenagers to do it for them, a lot of people in the older population this is a challenge for them” Provider 1, Workshop 2*
**Resources and infrastructure**	
Consumer perceptions: • Resources needed for full monitoring e.g. need devices, technical support, reliable connectivity, limited value if timeframe of monitoring is too brief • Lack of WiFi infrastructure, with limited coverage, particularly in regional areas and suburbs with blackspots • Need for back-up e.g. availability of emergency services on time to respond in case of device malfunction or gaps in monitoring	*“Is there someone at the other end monitoring the results if something happens – will they respond straight away? What if no one is around?” Consumer 2, workshop 2*
Provider perceptions: • Expensive for health systems to implement e.g. additional staffing requirements such as specialist doctors to read the data 24/7, supply of devices, additional infrastructure such as data storage, maintenance, upgrades • Care and regulation of equipment needed e.g. device malfunction, or machines may be dropped or broken, demand outstripping resources • Need admission pathways for inpatient care other than ED for lower acuity conditions • Technology issues e.g. stable and reliable internet access for patients across the catchment, streamlining and linking of data • Potential workload issues e.g. same amount of work for providers, but may increase workload for senior clinicians, increasing in-flow of ED patients if patients are concerned and do not understand the device alarms, volume of patients that need monitoring • Medico-legal issues around maintaining 24/7 clinician monitoring (which is not considered feasible by providers)	*“More admin staff are needed for chronic patients who don’t have someone that can help them. Their children have to take time off work to help them” Provider 2, workshop 2* *“Has already been trialled during COVID and found it required more admin staff to help the patients managing the platform” Provider 3, workshop 2* *“Looking at Rouse Hill, some areas don’t have the facility for this to be consistently up and running- in remote and older populations their technology might not hold… problems with NBN. This is a big challenge when the NBN goes out” Provider 6, workshop 2*
**Quality and safety of care**	
Consumer perceptions: • Contingency planning must be in place e.g. for technology failures or patient deterioration • Concerned digital monitoring will overtake face-to-face care • Validity and accuracy of monitoring • Relies on consistent patient follow up • Device security and data safety and privacy • Overreliance on technology for assessment of the patient and may miss something	*“How do I know this is the best care for my condition? Will it be overused by the healthcare staff because the lack of capacity in the hospital?” Consumer 1, workshop 6* *“Ability of doctors to pick up unexpected things will be limited” Consumer 1, workshop 8*
Provider perceptions: • Device or equipment malfunctioning • Legal ramifications if staff miss something, or if not contacted if an issue arises • Privacy and confidentiality concerns for staff and patients • Need to have safety processes in place in case an event occurs • Adverse events may be more readily picked up in inpatient settings • Qualifications and capacity of person at receiving end of monitoring data • Less control than when in hospital setting • Cannot do physical consults or assessments, less control than when in hospital setting, and less able to see all health cues without physical exam and “seeing” the patient • Not suited to all conditions, or all patients • Unreliability or other limitations of technology e.g. poor WiFi reception in some areas affecting communication, lack of trust in alerts, accuracy of devices and discrimination between genuine event and exertion in sport/exercise • Relies on patient compliance • Places responsibility and greater workload on senior doctors	*“What happens if the technology malfunctions?” Provider 5, workshop 2* *“if out in the community/outpatient and something happens—who is legally responsible?” Provider 1, workshop 6* “*Who is going to take ownership of the information? who will be responsible for it?” Provider 2, workshop 9* *“Where do they go if there is an issue? Not many options left for the specialist who was monitoring them or the technicians or physiologists, where do they get the patient reviewed if they notice something, there’s no path rather than being sent to emergency- this needs to be worked on and is a challenge- clinics are full and don’t have the service provision and people who do the monitoring don’t have a clinic” Provider 1, workshop 2* *“Could become a “cried wolf” situation – alerts are not trusted to be urgent and assumed to be a false alarm” Provider 7, workshop 6* *“There is no way for junior doctors to be involved in this model of care in the current system, thus this model increases workload for senior clinicians.” Provider 4, workshop 2*

## Discussion

**Fig. 3:**
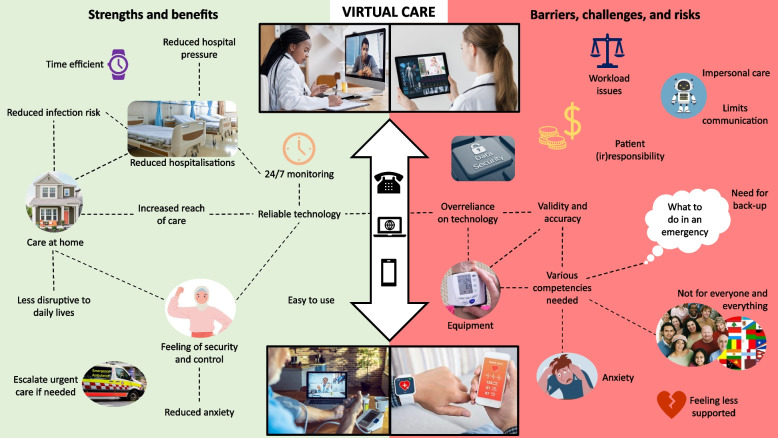
Summary of main findings

Our study elicited consumers’ and providers’ views and preferences about provision of care via virtual modes, proximally, to inform the design of a new metropolitan hospital, but with distal application to other similar settings nationally and internationally. A rich picture, summarising our main findings, is at Fig. 3. We found that virtual care was widely perceived to improve patient wellbeing, increase accessibility to care, and free up hospital resources. However, participants agreed that the model was not suitable for all patients or all conditions; minimum levels of health and digital literacy, and availability of reliable communication technology was considered essential, along with processes for escalating care if needed. Above all, choice in selecting the model, for both consumers and providers, was deemed paramount.

Virtual care has become increasingly common because of COVID-19, and published data on patient satisfaction with this mode of care is beginning to emerge. In a 2020 health department survey of 2,600 patients across NSW who had experienced virtual care provided by public hospitals during COVID-19 a high proportion rated the virtual care they received as ‘very good’ or ‘good’ and said, if given the choice, they would use virtual care again [34]. While respondents were predominantly positive about virtual care, as with our study participants, they preferred it was reserved for simple, straight forward consultations, routine appointments and referrals [34]. Clinicians across NSW were also recently surveyed about their experiences of providing virtual care [35]. Similar to the providers in our study, survey respondents generally perceived virtual care as an additional positive option that increases access and choice when used appropriately [35]. Health service evaluations [36–39] and academic literature [11, 14–16] also support the positive benefits of virtual care, including improved patient satisfaction and more personalised care.

Virtual care was perceived to be beneficial for diverse populations, improving access to care for those who could not easily visit hospitals, whether due to remote location, disability or restricted access to transport. It was also perceived to be beneficial for CALD populations, provided adequate interpreter services were provided. As in our study, focus testing by others found that CALD populations supported virtual care, provided interpreting services were integrated into the model [35].

Concerns raised by consumers included provision of back-up options in case of technology failure and development of clear escalation processes in case more urgent care was needed (especially outside normal working hours). These apprehensions are timely: a recent review on patient safety associated with delivery of care via virtual modes found that patient risks associated with telehealth are not well understood or addressed [40]. Concerns raised by providers about the limitations of virtual media, such as not being able to physically examine the patient, are also evident in the literature. For example, a recent US study found blood pressure was only measured in 1 in 10 virtual care consultations in comparison with 7 in 10 face-to-face visits [41]. Providers in our study expressed concerns about the medicolegal ramifications of missing a diagnosis, and studies that have found errors and inappropriate referrals for some conditions support these concerns [4].

When implementing virtual care models, consideration should be given to addressing identified barriers such as availability of information and communication technology infrastructure, and usability of the system for both clinicians and consumers. Considering consumer and provider views when designing new models of care has been shown to improve uptake [42]. While usability studies have strong potential to improve adoption and safety of virtual care delivery modes [43], there is limited research on how usability evaluation has been used to support design and implementation of telehealth and virtual care systems more broadly [44, 45].

The perception, voiced by both consumers and providers, that virtual care should be considered as a supplementary mode of care to support other modes, leads to the concept of blended models. In the consumer and provider consultations, participants suggested virtual care be blended with face-to-face care, rather than with other innovative models. In the literature, however, virtual care was more commonly blended with other models or forms of care, namely hospital in the home, integrated care or digital hospitals. While blended models are more likely to facilitate patient choice, and appear to produce superior outcomes in some studies [9, 13, 46], it may be critical to assess whether the blending of models increases, decreases, or shifts resource requirements. When blending integrated and virtual care, for example, one review found that there were increases in outpatient clinic visits and patient-initiated telephone contact for those receiving integrated telemonitoring, as well as increased nurse time, contacts, and visits [23].

The ability of the model to reduce pressure on hospitals may be more a perception on the part of consumers than reality. While some studies have found reduced readmissions associated with virtual care [17], others have shown mixed results [14, 18, 21, 47, 48]. Inpatient hospital beds are typically costed on the number of clinical staff needed, rather than the number of physical beds, and it is likely than any staff freed up from face-to-face care will be needed to deliver care via virtual modes. Studies have found, for example, that virtual care results in higher healthcare utilisation for some conditions [49]. The grey literature also reports that implementation of virtual care can be hampered by change resistant workplace cultures and organisational leadership, and that this can result in variable use and uptake amongst clinicians [50].

### Limitations

As the study was conducted primarily via Zoom, it is likely that there was a positive bias toward those who prefer, or are more comfortable, with virtual care delivery modes. The Australian Digital Inclusion Index (ADII) [51] is a composite measure that scores access, affordability and digital ability over a range of 0–100. The threshold for inclusion (ADII 61 and above) indicates that a person scoring above that level can make accessible, affordable, and effective use of the internet. For our participant community in 2021, the ADII ranged between 71.0 (Included) for the Southern part of the catchment and 80.0 (Highly Included) for the Northern portion [51], so it is likely that our results are reflective of the broader community. Additionally, the consultations were conducted with residents and providers of one local health district in metropolitan Australia. We did not explore whether participants had previous experience of virtual care, and this may have influenced their responses. Finally, non-English-speaking participants were of largely of Chinese descent (13% of consumers). Other non-English speaking participants were invited to contribute to consultations, but low participation rates were observed.

## Conclusion

Virtual care modes are positively perceived by broad range of consumers and providers and have unrealised potential to be an important part of innovative hospital care. Through enhancing patient choice and agency, virtual care can position the patient at the centre of clinical decision-making. A successful virtual care model would require sufficient infrastructure, including connected technology and clinical spaces from which to deliver care, training on care delivery modes for both consumers and providers, and clinical governance frameworks to manage data security and deliver care safely. Implementation of this mode of care requires careful consideration of resources, however; these include resources to support learning of new skills for consumers and providers, provision of clinical escalation processes, provision of skilled clinical staff, adequate and reliable integrated communication technology, and integration with other hospital and health services.

## Supplementary Information

**Additional file 1.** Consumer focus group script.

**Additional file 2. **Provide focus group script.

## Data Availability

All data generated or analysed during this study are included in this published article [and its supplementary information files].

## Abbreviations

CALD: Culturally and linguistically diverse
CKD: Chronic kidney disease
COPD: Chronic obstructive pulmonary disease
ECG/EKG: Electrocardiograms
EOI: Expression of interest
GP: General practitioners
LHD: Local Health District
NSW: New South Wales
PICF: Participant Information and Consent Form
