# Supplementary material for: The diversity of providers’ and consumers’ views of virtual versus inpatient care provision: a qualitative study
Source: BMC Health Serv Res. 2023 Jul 4;23:724. doi: 10.1186/s12913-023-09715-x (PMC10318821; doi:10.1186/s12913-023-09715-x)
Supplement: Supplementary file 1 — Additional file 1. Consumer focus group script. [file 12913_2023_9715_MOESM1_ESM.docx]

**Consumer: General Welcome**

“Thank you for coming along today and participating in our study. My name is XXX.

Acknowledgement of Country

The purpose of this workshop is to capture the needs of the community and their perspectives on new ways of delivering acute care. What we talk about today will inform the development of the new hospital. We really appreciate your time and thank you very much for being here today.

**Consumer stream:** As consumers of healthcare, your experiences as patients are very valuable. However, please note that there is no obligation to disclose details of personal healthcare issues.

Throughout the workshop a researcher/facilitator will be with each table group to take notes, facilitate discussion and ask you questions. The workshop will go for 2 hours with a 10-minute break with refreshments after the first hour.”

You will remain unidentifiable in the analysis and write-up of any findings relating to this research.

If you have any questions throughout the day, please ask one of the xx University staff members (**introduce all staff members**).

**Group Introduction**

“Good afternoon (morning/evening etc.) and welcome to the workshop. Thanks for taking the time to talk with us about what you would like to see in the new hospital. My name is (**insert name here**), and I am from the xx Institute at xx University. My role as moderator will be to guide the discussion today.

Please note that there are no right or wrong answers but rather differing points of view. Please feel free to share your point of view even if it differs from what others have said. You don’t need to agree with others’ opinions, but we ask that you listen respectfully as others share their views.

We are taping the session because we don’t want to miss any of your comments. People often say very helpful things in these discussions, and we can’t write fast enough to get them all down. However, to make the recording as clear as possible, we ask that only one person speak at a time. And to remind you, no-one is identifiable on the recording.

We will be on a first name basis today, but we won’t use any names in our reports.”

**“**We would like you to reflect on a patient or patients who may or may not benefit from different “models of care”. Today, we will talk about virtual care in our workshop today. We are interested in your ideas and would like you to reflect broadly as well as on your experience as a person who may or may not benefit from integrated care.

**Virtual Care**

Patient care and consultation delivered through telephone or video communication

SHOW image: Virtual Care

Ivy is a 40-year-old woman who developed chest pain along with an irregular heartbeat following a dental procedure. She visited the local Emergency Department where no abnormality was found and was discharged. As she was still concerned about a sudden heart attack, she was fitted with a digital heart monitor with chest leads that talked to an application on her smart watch. Ivy was shown how to indicate an unusual heart event using her watch. Anytime Ivy tagged an event, the information was sent to a health care professional at the moment it happened.

**We would like to ask questions from Ivy’s as well as your own perspective. Let’s start with Ivy:**

**From Ivy’s perspective:**

1. **What is good about this model for Ivy?**
2. **What about this model might make it difficult for Ivy?**

*Additional prompts*

Can you think of anything about it that might be impractical?

Can you think of anything about it that might be unachievable?

1. **What needs to be in place for this to work for Ivy?**

**For example, systems, processes, people, skills and equipment?**

**Now from your perspective:**

1. **What about this model might be good for you and your family?**
2. **What about this model might make it difficult for you and your family?**

*Additional prompts*

Can you think of anything about it that might be impractical?

Can you think of anything about it that might be unachievable?

1. **How easy is this to use for you?**
2. **What would stop you using it?**
3. **Can you think of other people who would have difficulty using this model?**
4. **We have already asked for Ivy but what other things needs to be in place for this to work for you?**

**For example systems, processes, people, skills and equipment**

**General questions:**

1. **Is there anything about the model that concerns you?**
2. **Can you see any safety issues for yourself?**
   - Why is that?
   - *Can you suggest a better way?*

*(Additional prompts)*

*Are there any potential risks that you can identify?*

1. **What other illness and injuries might this model work for?**

**Concluding remarks:**

We will be looking at your data to find commonalties between consumers.

We are conducting exploratory research to gather information only. Thank you for your time.

**Note: All images were publicly available and downloaded from Google.**
